# Shape detection beyond the visual field using a visual-to-auditory sensory augmentation device

**DOI:** 10.3389/fnhum.2023.1058617

**Published:** 2023-03-02

**Authors:** Shira Shvadron, Adi Snir, Amber Maimon, Or Yizhar, Sapir Harel, Keinan Poradosu, Amir Amedi

**Affiliations:** ^1^Baruch Ivcher School of Psychology, The Baruch Ivcher Institute for Brain, Cognition, and Technology, Reichman University, Herzliya, Israel; ^2^The Ruth and Meir Rosenthal, Brain Imaging Center, Reichman University, Herzliya, Israel; ^3^Research Group Adaptive Memory and Decision Making, Max Planck Institute for Human Development, Berlin, Germany; ^4^Max Planck Dahlem Campus of Cognition (MPDCC), Max Planck Institute for Human Development, Berlin, Germany; ^5^Weizmann Institute of Science, Rehovot, Israel

**Keywords:** spatial perception, visual-auditory, sensory substitution, sensory substitution device (SSD), visual-spatial perception, auditory spatial perception, multisensory spatial perception, multisensory perception

## Abstract

Current advancements in both technology and science allow us to manipulate our sensory modalities in new and unexpected ways. In the present study, we explore the potential of expanding what we perceive through our natural senses by utilizing a visual-to-auditory sensory substitution device (SSD), the EyeMusic, an algorithm that converts images to sound. The EyeMusic was initially developed to allow blind individuals to create a spatial representation of information arriving from a video feed at a slow sampling rate. In this study, we aimed to use the EyeMusic for the blind areas of sighted individuals. We use it in this initial proof-of-concept study to test the ability of sighted subjects to combine visual information with surrounding auditory sonification representing visual information. Participants in this study were tasked with recognizing and adequately placing the stimuli, using sound to represent the areas outside the standard human visual field. As such, the participants were asked to report shapes’ identities as well as their spatial orientation (front/right/back/left), requiring combined visual (90° frontal) and auditory input (the remaining 270°) for the successful performance of the task (content in both vision and audition was presented in a sweeping clockwise motion around the participant). We found that participants were successful at a highly above chance level after a brief 1-h-long session of online training and one on-site training session of an average of 20 min. They could even draw a 2D representation of this image in some cases. Participants could also generalize, recognizing new shapes they were not explicitly trained on. Our findings provide an initial proof of concept indicating that sensory augmentation devices and techniques can potentially be used in combination with natural sensory information in order to expand the natural fields of sensory perception.

## Introduction

In humans, vision is unequivocally considered the dominant sense (Colavita, 1974; Hutmacher, 2019). In addition, cumulative evidence has demonstrated that vision tends to dominate the perception of spatial location when presented alongside conflicting information from other senses. This phenomenon is demonstrated by the well-known ventriloquist effect (Bruns, 2019). A further connection between the senses of vision and audition is demonstrated in the McGurk effect, in which changing visual stimuli impact the auditory stimulus understood to be heard (McGurk and MacDonald, 1976). And yet, the human visual field has a limitation in that it spans 210° (Traquair, 1938; Strasburger, 2020), leaving humans with visual-perceptual blind spots. In addition, a large part of the 210° field of view is peripheral vision (Millodot, 2014), which undergoes a dramatic spatial/temporal discontinuity (Shapiro et al., 2010). On the other hand, the human auditory spatial field encompasses the entire 360° range. As such, our perception of space largely depends on the integration of information from these two crucial senses.

Irrespective of our perception of them, material objects in space are located around us and known to “have an intimate relationship with space” (Casati and Varzi, 1996, p.205). We are constantly tasked with reliably identifying the location at which these objects around us are found. This is where the integration of the senses and multisensory interactions are thought to come into play. It is known that multisensory integration is an acquired process (Gori et al., 2008) and that adults continually update their perceptual systems, calibrating them to their sensory circumstances (Ernst, 2008). Previous studies have taken different approaches as to how observers can recognize visual shapes from auditory cues (Bach-y-Rita et al., 1969; Bach-y-Rita, 1983, 2004; Ptito et al., 2005; Amedi et al., 2007; Striem-Amit et al., 2012; Maidenbaum et al., 2014). There is still an ongoing debate about how vision and audition are integrated for stimuli learned in the adult brain (e.g., Hertz and Amedi, 2015). Prior research has even indicated that cross-modal attenuation (deactivation) can reverse in sensory cortices after training on sensory substitution algorithms, and associative areas can change their sensory response profiles (Hertz and Amedi, 2015). Research indicates multisensory interactions are found in many cortical and subcortical locations (Alais et al., 2010). This considered, the goals of the present study are first and foremost pragmatic, exploring whether and to what extent sighted people can integrate auditory and visual stimuli presented in 360° into a coherent percept.

We perceive the space around us and understand it through shapes and objects. In this respect, shape recognition has been studied widely with visual objects (Milner, 1974; Pietrini et al., 2004; Peelen and Kastner, 2014; Erdogan and Jacobs, 2017). However, when addressing the role of audition in shape perception complementary to or substituting for vision, it has been shown that both sighted and blind observers can process the spatial properties of objects or shapes (Carello et al., 1998; Collignon et al., 2009; Bizley and Cohen, 2013). For example, audition alone can provide information regarding shape curvature (Boyer et al., 2015). In this study, we examine the abilities of sighted people to recognize visual shapes from hearing in a 360° space around their heads, unlike in the studies above, in which shapes are perceived only in the frontal visual field.

The current study utilized the EyeMusic algorithm, a sensory substitution technique that uses a left-to-right sweep-line technique that processes the visual image column by column (Abboud et al., 2014), in combination with spatial audio (Ambisonics), to create a 360° perceivable version of the algorithm named “Vision360.” The resulting auditory-rendered musical fragments preserve the image’s shape and spatial positioning. In this study, we tested the possibility of combining spatially oriented sensory information to form single or multiple shape percepts while receiving information beginning in the visual field and ending in the auditory field.

We employed the aforementioned procedures to test whether participants without sensory limitations can identify a visual shape or sets of shapes presented to them in a 360° azimuthal orientation around them, thereby building upon auditory perception for enhancing their natural visual field. Moreover, we asked whether individuals would be able to integrate non-simultaneous partial visual and auditory information extrapolated into a single 360° image. According to our predictions, utilizing the auditory modality to augment the limitations of the visual system spatially will shed light on sighted participants’ abilities to extend their perception beyond the natural range and perceive spatially dispersed visual information. This ability has not been previously tested using visual-to-audio SSDs. In addition, the results will provide insight into utilizing 360° audio cues to expand the normal SSD range from a 2D image to the surrounding 3D space. We also wish to test such a system’s impact on shape recognition and generalization to untrained visual shapes. Finally, we discuss several practical and more theoretical neuroscience-based future directions following this approach.

## Materials and methods

### Participants

A total of 15 participants (6 women, aged 28.5 ± 5.8 years) with no known neurological or sensory impairments participated in the study. All participants reported normal or corrected-to-normal hearing and vision. The institutional review board (IRB) of the Reichman University approved the study. All participants were recruited *via* social media and signed an informed consent form. They were provided 40 nis per hour compensation for their participation in the study and had no prior familiarity with the EyeMusic device, the algorithm, or any other SSDs. In determining an appropriate sample size, we followed along the lines of a previous study conducted by our lab as a proof of concept introducing the novel EyeMusic algorithm, on which the current algorithm is based. The study by Abboud et al. (2014) was conducted on 12 participants.

### Apparatus

**FIGURE 1:**
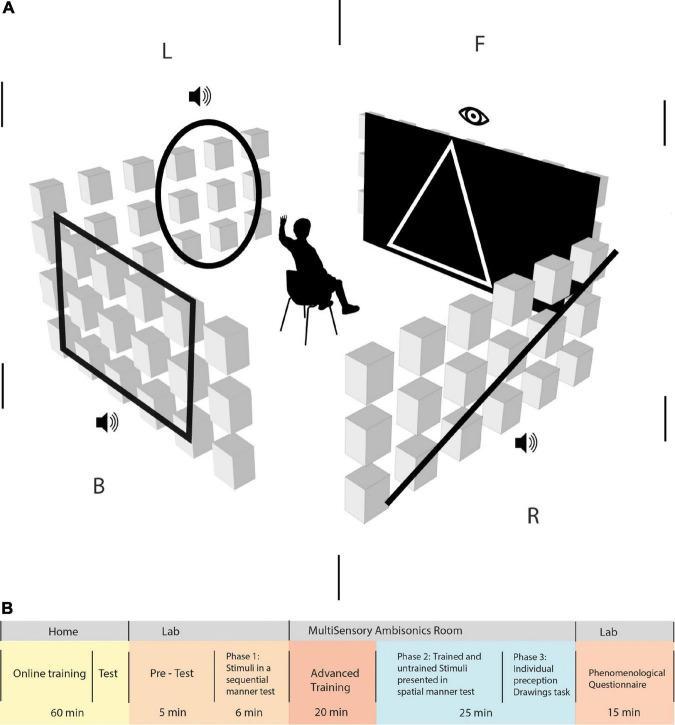
**(A)** Simulation of the Vision360 experiment in the multisensory room at the lab. The participants sat in the middle of the room, 2 m away from each wall, at an ear height of 1.4 m. An ambisonic system operated 21 speakers on every wall, with the corner speaker column shared among adjacent walls. F | R | B | L represents the division of space to front, right, back, and left, respectively, in an egocentric manner (in relation to the participant’s body and position). Participants were required to focus on the front side of the room, where they perceived a visual stimulus and then an auditory stimulus. It appeared only on the participants’ right, back, and left sides, in this order. Trained EyeMusic shapes and untrained shapes are perceived as stimuli both visually and auditorily. **(B)** Study outline. Participants went through various phases to complete the experiment: 60 min of basic training on the EyeMusic SSD at home. Following this, participants who passed the online training test at home were invited to the experiment performed at the lab and in the MultiSensory Ambisonics room. Upon the arrival of the participants to the lab, they went through a 5-min pre-test on the EyeMusic SSD material before moving on to phase 1 of the experiment. Phase 1 of the experiment was a test of stimuli presented sequentially for 6 min. Then, they moved on to the advanced training phase, where stimuli were presented to them in the ambisonics system for 20 min. Phase 2 of 25 min was a test that consisted of trained and untrained stimuli, which was presented spatially in the room. Then, they moved to phase 3, where participants were asked to draw the stimuli they perceived, and finally, they took a phenomenological questionnaire.

The study took place in a cube-shaped soundproofed room, 408 cm (length) × 400 cm (width) × 268 cm (height) in size. A total of 72 loudspeakers were arranged along the walls in three horizontal rings at the following heights: 48, 148, and 248 cm, with an even azimuthal spacing of 15° among each adjacent pair (Figure 1A). Furthermore, 25 additional speakers were mounted on the ceiling in a 5 × 5 grid. All speakers in the room were measured and calibrated for spectral and delay matching. Participants were placed in the center of the room, with their heads at the height of the middle speaker ring. The center point was calibrated to a height of 148 cm (the level of the middle horizontal axis on the wall). The study was operated from a separate soundproofed control room. Interaction with the participant was carried out using a talkback microphone system and camera. The auditory stimuli were played from a local PC. Multichannel digital-to-analog conversion utilized a Dante network in combination with 13 Crown DCi 8| 300DA network-enabled amplifiers. The visual projection was performed using a sound transparent screen and a projector calibrated to fill between −45° and 45° of the front-facing azimuthal angle (the entire front wall), enabling synchronized visual and auditory stimuli.

### 360° audio-visual transformation

Visual-to-auditory transformation of the current study, Vision360, is based on techniques of the EyeMusic technology (Abboud et al., 2014), in which pixels along the *Y*-axis (bottom to top) are converted to consecutive pitches along a pentatonic scale. The Vision360 algorithm takes a similar approach to vertical pixel positioning to pitch conversion while adding a spatial audio component, enabling the position of the sound source to arrive at any azimuthal position (360°) encircling the person. To maintain coherence with the original EyeMusic (in which temporality and spatial positioning are linked) and the sweep direction remains constant, in the present study, we transform the left-to-right sweep-line into a clockwise azimuthal-sweep surrounding the person. Each white-colored pixel is converted into a MIDI note pertaining to its location in the image, creating a MIDI file that can then be played back at a chosen speed. The image ratio used for all stimuli was 180 × 30 pixels (180 horizontal and 30 vertical), where the *X*-axis is understood to contain the full 360° ring surrounding the participant (e.g., if *X* = 0 is set to the front center of the room, then *X* = 90 will be at the back center of the room). The experiment was programmed as a Max MSP patcher, utilizing the Spat5∼ library for Higher Order Ambisonics (HOA) encoding and decoding. Ambisonics is a mode of recording and reproduction based on a representation of the sound field excitation as a decomposition into spherical or circular harmonics, respectively. This achieves a physically accurate sound field reproduction restricted within a designated spot in the center of a loudspeaker array.

### Stimuli

In the present study (that included only seeing individuals), all images contained white pixels with a black backdrop. All stimuli in their entirety lasted 10 s, with a proportional division of space converted to time (i.e., visual 2.5 s, audio 7.5 s). Each visual/auditory pixel was representative of ∼2° of the surrounding space. All stimuli used a pleasant-sounding timbre created using simple additive synthesis (combined sine tones based on a fundamental frequency) and its overtones (whole number multiples of the fundamental frequency) at lesser amplitudes. Playback was set to a comfortable hearing level (SPL ∼65 dB). All pitch frequencies used were between 49 and 3,135 Hz.

### Vision360 stimuli

Vision360 stimuli were a combination of a visual projection followed by spatialized audio. These stimuli started as a visual projection onto the frontal 90° screen (between −45° and 45° azimuth, 40 × 30 pixels; vertical range between 30° and −28°), then the projected visuals disappeared and were followed by the remaining 270° presented through spatially moving sound. The virtual sound source moved in a clockwise manner, beginning at the 45° azimuthal point and commencing to encircle the participant fully.

### Basic at-home training

**FIGURE 2:**
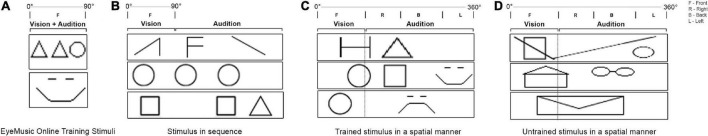
Various types of stimuli used during the experiment. **(A)** Images used during the online training. Participants see the visual image and simultaneously hear the monophonic audio rendition that represents this image. **(B)** Stimulus in sequence, participants perceived in their front 90° a visual shape and afterward heard the rest of the auditory stimulus in headphones. **(C)** Stimuli with three shapes in sequence presented 360° around the participant. The front is visually projected, while the sides and back are rendered as spatial audio. **(D)** Unified audio-visual stimuli presented partially in visual and partially in audio.

Following recruitment, participants underwent approximately 60 min of home training (for study outline, see Figure 1B). They trained on monophonic renditions of stimuli from home, using headphones and a website platform created for the experiment. Home training included nine lessons, each including several images. Clicking under the images activated audio representations of the image generated by the EyeMusic algorithm. Each lesson was followed by a short quiz, including five multiple-choice questions, to give feedback to participants. Training consisted of simple geometric shapes such as a square, triangle, circle, horizontal/vertical/diagonal lines, arrows, simple house, happy/sad/indifferent face, and “F”/“H”/“E”/“N” letters (see Figure 2A). At the end of the home training, participants received a final test containing 10 Alternative Forced Choice (4AFC) questions and needed a score of 70% to pass to the subsequent phases of the experiment. Participants who passed were invited to the lab within 7–15 days after completing the home training. All participants who were invited to learn the algorithm at home succeeded to pass the test, except for one who did not complete the home training and did not take the test.

### In laboratory pre-test

Once they arrived at the lab, participants received a brief explanation about the experimental space. Then they underwent a ∼5-min review of the EyeMusic algorithm. Participants then retook the final test from home training (including ten 4AFC questions) to ensure comprehension of basic EyeMusic principles learned during the online training. This test was deemed necessary because participants in the experiment arrived at the lab 5–7 days after passing the online training. Thus, it was essential to validate that the material was well remembered before moving forward to subsequent phases of the experiment conducted physically in the lab. Participants could also choose to retake this test while in the lab to ensure comfotability with the learned material. Seven participants took the test twice consecutively, the rest took it once.

### Shapes in sequence: Phase 1

In the first experimental phase (phase 1), participants were seated in the center of the experiment room facing forward and asked to fixate their gaze on a 5 × 5 cm red square placed at the 0° azimuth and 0° elevation points. Each stimulus was composed of two to three shapes, starting with a visual shape presented on the front screen, which disappeared, followed by two separate shapes played through headphones (see Figure 2B). Audio given during this stage was a monophonic rendering and played equally to both ears. All shapes shown during this phase were familiar to the participants from prior training. The test included ten stimuli. Each stimulus was presented only once, starting and ending with a cue sound from the ceiling to notify the participants when a new stimulus was about to start and after it had ended. Stimuli from this phase are referred to as “shapes in sequence.” Following each stimulus, participants were presented with Four-Alternative-Forced-Choice (4AFC) questions. Each possible answer displayed three shapes in chronological sequence from which they had to identify the one they were presented with (i.e., the sequence simulated the order of presentation; the left shape was always the visual shape).

### Advanced training

Before the second phase of the experiment, participants underwent another training session of approximately 20 min. During this session, participants were introduced to the Vision360 transformation, then presented with 18 stimuli. The purpose of the training was to introduce the participants to the 360° audio abilities of the room and let them adjust to its immersive nature. As before, participants were asked to fixate their gaze. In this phase, some stimuli were similar to those the participants had already learned, while others were new (untrained). During training, participants were given feedback and told if their responses were correct or incorrect. If an answer was incorrect, the stimulus was repeated. Participants could ask to repeat the stimuli as many times as they needed. Performance was assessed with 4AFC questions.

### 360° audio representation: Phase 2

During phase 2 of the experiment, participants were tested on 22 Vision360 stimuli. Of the 22 stimuli, 11 consisted of familiar shapes, beginning with a visual shape on the front screen and then the additional 1–2 shapes presented through audio at different locations in the 360° space (see Figure 2C). These 11 stimuli included previously untrained shapes and consisted of long extended shapes played throughout the 360° surrounding space (see Figure 2D). These stimuli could consist of two familiar shapes or new ones being played simultaneously. Stimuli could also include shapes presented as truncated at the edge of the screen and completed in audio. All stimuli were followed by a 4AFC question depicting the 360° stimulus as one of the choices. Other possible responses contained different (yet similar) shapes to the original at similar positioning. No feedback was provided to participants following their responses. Phase 2 included a 5-min break.

### Drawing 360° images: Phase 3

**FIGURE 3:**
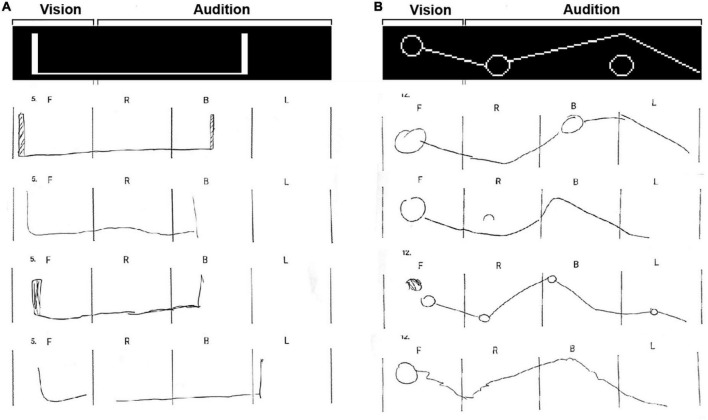
Two examples of the drawing task stimuli with 8 participants’ drawings. **(A)** Example of expanded single shapes type of stimuli. The group score for the number of shapes was 85 ± 24.6%, the score for the accuracy of the shapes themselves was 83 ± 24.4%, the group score for the proper positioning of the shapes was 86.7 ± 29.7%, and the group score for the unified visual-auditory shape was 70 ± 45.5%. **(B)** Example of a stimulus from category 3 (combining expanded and trained shapes in tandem). The group score for the number of shapes was 77.7 ± 10.3%, the score for the accuracy of the shapes themselves was 43.7 ± 17%, the group score for the proper positioning of the shapes was 66 ± 14.6%, and the group score for the unified visual-auditory shape was 65.3 ± 36.6%.

Finally, participants were presented with 13 additional Vision360 stimuli, which they were asked to draw in their entirety (i.e., composed of both visual and auditory segments) on a piece of paper handed to them before the task. A few examples of participants’ drawings are presented in Figure 3 (for the rest of the drawings, see Supplementary material). These 13 stimuli were divided into the following three categories: category 1 included six stimuli, each containing 2–4 trained shapes placed at different locations; category 2 included two stimuli, each containing a single extended shape meant to test whether participants experienced the auditory and visual information as unified; and category 3 included five stimuli, each containing a combination of an extended shape along with smaller trained shapes in tandem. Assessment of the drawings was done by counting and rating by (1) quantity: the number of shapes drawn; (2) shape recognition: whether the shapes in themselves were correctly identified; (3) positioning: whether the shapes were placed in the proper positions; and (4) unifying audio-visual: whether the shapes broken between visual and audio were drawn connected as a single shape. Separate average scores were given for each group of stimuli.

For *category 1* (stimuli containing separated trained shapes), participants were scored in all four measures (quantity, shape recognition, positioning, and unifying audio-visual). The number of shapes drawn was compared to the number of shapes in the original stimuli. A point fraction was deduced for any shape added to or missing from the stimulus (e.g., if someone drew 2 or 4 shapes in a stimulus that had three shapes altogether, they would receive 2/3). Participants were also scored on the correctness of the shapes. Points were given for every correct shape and divided by the number of shapes (as above). Positioning scores were given based on whether participants correctly located the shapes within or between the F| R| B| A brackets. Scores were calculated in the same manner as above. Two of the five stimuli in this category had a shape broken between the visual and auditory fields. We tested whether participants recognized the shape as unified and scored with a binary rating (1 or 0). A unified shape would contain a continuous drawn line going over the “F| R” brackets).

In *category 2* (expanded single shapes), the number of shapes drawn was compared to the number of shapes perceived in the original stimuli. In shape recognition, a point fraction was subtracted if they did not unify the shape (breaking the drawn line between the “F| R” brackets). The position of these shapes was rated according to their location reflected in the brackets. Unifying audio-visual was rated in a binary rating in the same manner as in the category above.

*Category 3* (combining expanded and trained shapes in tandem) was scored similarly to category 1.

The group score for each of the measures within each category was calculated by summing each participant’s stimuli scores within the measure, then averaged among the group and converted to percentages (see Supplementary material for participant results).

### Questionnaire

The phenomenological questionnaire was based on a questionnaire from the study by Buchs et al. (2021). It included questions regarding the perceived learning and perceived difficulty in each experiment stage and the pleasantness of the stimuli.

### Verbal interview

After completing the study, we conducted a verbal interview with each participant to assess more accurately the participants’ subjective experiences. They were asked to freely describe their experience, their perception of the shapes and whether or not they closed their eyes. Further questions pertained to experienced unification of shapes that began visually and continued auditorily. Finally participants were asked regarding their overall experience, beginning with the online training and commencing with the last task of the experiment.

### Statistical analysis

All statistical analyses were performed using JASP (version 0.25). Wherever relevant, *p*-values reported in the results were corrected for multiple comparisons. All significant *p*-values remained significant after correction. To assess whether our experimental, control, trained, and generalized conditions displayed above chance level mean correct response (MCR), we performed a tailed one-sample Wilcoxon test against an alternative mean of 25%. To assess whether subjects improved between conditions, we performed a two-tailed Wilcoxon signed-rank test between conditions.

## Results

**FIGURE 4:**
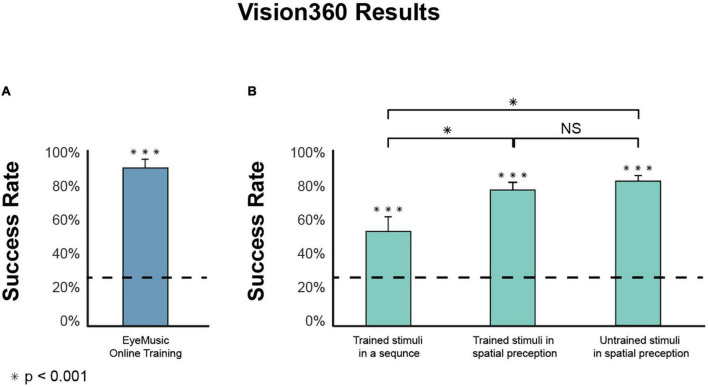
Experiments results. **(A)** Participants in the EyeMusic online training had a statistically significant success rate in the final test of 89.3 ± 5.5% [mean correct response ± SD; **(A)** bars indicate the standard error; dashed line indicates chance level]. **(B)** Experimental phase tasks’ results are divided into three categories: Recognition of stimulus in a sequence (54.6 ± 16.8%), the success rate of spatially perceiving trained shapes (78.8 ± 12.2%), and recognition of untrained shapes (Generalization) perceived spatially (82.4 ± 14.3%). There was no significant difference between the trained and generalized conditions (W(14) = 22, *p* = 0.349). However, between the trained sequentially presented stimulus and the trained stimuli presented spatially, there is a significant difference (W(14) = 117, p_corr_ < 0.01), as well as between the stimuli in a sequence compared to the Generalization of stimuli presented spatially (W(14) = 120, p_corr_ < 0.001). ***Means significantly above chance. *Means significantly different between two conditions. NS, not significant.

*Participants can successfully learn the EyeMusic algorithm using a brief online protocol*. The percent of correct responses in the online test stood at 89.3 ± 5.5% (mean ± SD; Figure 4A), revealing a high rate of correct answers, significantly above chance level (W(14) = 120, *p* < 0.001).

*Participants performed better in a spatial task than in a sequential task*. The success rate for shapes in sequence stood at 54.6 ± 16.8% and was significantly above the chance level (W(14) = 120, p_*corr*_ < 0.001), showing that participants recognize stimuli composed of both visual shapes and monophonic audio-rendered fragments in sequence (Figure 4). When participants underwent a similar task under the same temporal condition in a 360° space, they had a correct response rate of 78.78 ± 12.2%, significantly higher than chance level (W(14) = 120, p_corr_ < 0.001) as well as higher than perceived stimuli in a sequence (W(14) = 117, p_corr_ < 0.01).

*Using Vision360, participants were better at recognizing generalized stimuli than stimuli presented sequentially in monophonic rendering*. We tested the participants’ ability to generalize shapes received in 360°. Participants had a correct response of 82.4 ± 14.3% with a chance level of 25% (W(14) = 120, p_corr_ < 0.001). Participants were better at recognizing untrained stimuli in 360° than trained stimuli presented sequentially monophonically (during phase 1), with significantly higher results (W(14) = 120, p_corr_ < 0.001).

*Participants successfully recognized both trained and untrained stimuli to a similar extent*. To compare the abilities of the participants to recognize and orient the trained vs. untrained stimuli in Vision360, we performed a paired Wilcoxon test between the two conditions, which found no significant differences (W(14) = 22, *p* = 0.349). Meaning participants were successfully able to perform generalization.

**FIGURE 5:**
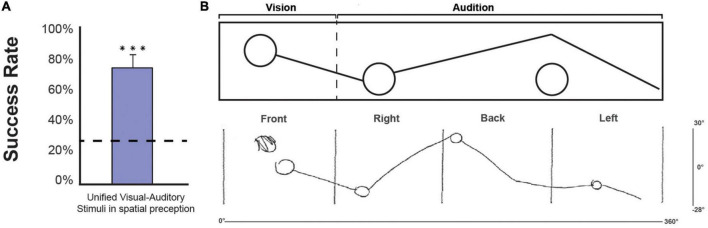
**(A)** Group result for shapes that began in the visual field and continued auditorily. We performed a one-tailed one-sample Wilcoxon test against chance. The correct response rate was 76.6 ± 15.1%, significantly higher than the chance level (W(14) = 120, *p* < 0.001) (bar indicates the standard error; dashed line indicates chance level). **(B)** An example of a full visual-auditory stimulus as processed in the Vision360 application. The “vision” section of the stimulus is perceived by the participants in the front, and the “Audition” section is perceived by the participants auditorily (starting from left to right in relation to the participants’ location). Underneath the stimulus, an example of a drawing by participant number 9, taken from the drawing phase of the experiment is shown. Front | right | back | left are the space expressions standardizing the division of space for the participants according to their egocentric position in space. The *x*-axis of 0°–360° represents the horizontal coverage of the stimulus in space, and the *y*-axis of 30°–28° represents the vertical coverage of the stimulus in space. ***Means significantly above chance.

*Participants can unify shapes presented spatially, where they begin visually and end auditorily* (Figure 5). The correct response rate for the 13 stimuli, which included shapes that started in the visual field and continued auditorily, was 76.6 ± 15.1%, significantly higher than the chance level (W(14) = 120, p_corr_ < 0.001). This finding indicates that participants can correctly unify shapes composed of visual and auditory components perceived in 360°.

Phase 3 consisted of 13 drawing tasks. In the drawing task, participants were asked to draw what they perceived both visually and auditorily. A few examples of different participants’ drawings are presented in Figure 3 (for the other participants’ drawings, see Supplementary material).

*Category 1* (stimuli containing 2–4 separated trained shapes), the group-averaged accuracy of perceiving the number of shapes was 85.6 ± 13.2%, for shape recognition was 58 ± 16.2%, for proper positioning was 78 ± 13.4%, and for unifying audio-visual was 46.7 ± 41.4%.

*Category 2* (expanded single shapes), the group-averaged accuracy of perceiving the correct quantity of shapes was 85 ± 24.6%, for shape recognition was 83 ± 24.4%, for proper positioning was 86.7 ± 29.7%, and for unifying audio-visual was 70 ± 45.5%.

*Category 3* (combining expanded and trained shapes in tandem), the group-averaged accuracy of perceiving the correct quantity of shapes was 77.7 ± 10.3%, for shape recognition was 43.7 ± 17%, for proper positioning was 66 ± 14.6%, and for unifying audio-visual was 65.3 ± 36.6%.

### Verbal interviews

In the verbal interviews, 8 out of 15 participants stated that spatial information helped them recognize and remember the shapes. Participant #12: *“The different locations of the shapes has helped me to remember them, whether a specific shape had appeared from the right or the left*”; Participant #3: *“I imagined the shapes in a way that they would immerse me around my body, then I performed some kind of flattening of the space around me to a strip. After I drew it, I would rethink it and correct the locations if needed, using my memory”;* Participant #9: *“I paid attention to the size of the room; I heard it on the left and not only in the back, so when I wasn’t sure what shape it was I used the sides, it was helpful.”* Out of 15 participants, 14 reported experiencing the shapes passing between visual and auditory as intuitively unified. Participant #10: “*It depends on the location of the sound. If the visual shape was cut by the end of the screen on my right, and that’s where exactly the sound had appeared right after, I understood it is connected*”; participant #3: “*I think that every time I saw the visual shape halved by the end of the screen to my right, I expected that it would be completed and continued with some kind of sound.”* Out of 15 participants, 7 indicated using their index finger to trace the auditorily received visual shapes through the air. Most of them stated they did it to help them recognize the perceived shapes. Participant #14: *“I drew with my fingers in order to recognize the shapes”*; Participant #15: “*I used my fingers to physically draw and imagine the shapes, also sometimes I did close my eyes to imagine the shapes.”* Out of 15 participants, 7 closed their eyes while experiencing some of the auditory cues, stating it helped them focus on recognizing the shapes when experiencing the auditory part of the stimuli. During phase 2, participants said they were replaying melodic memory and recalling mental imagery to answer the different phases. Participant #5: *“I tried to neutralize my visual sense, and I felt it strengthened my auditory sense. When I closed my eyes, it helped me imagine what I heard.”*

## Discussion

The current study investigated the role of auditory spatial perception in recognizing visual geometric shapes presented in a 360° space. To achieve this, participants needed to combine visual information with auditory information conveyed through a visual-to-auditory SSD. We asked participants to detect and orient shapes by reporting their egocentric spatial location (Front/Right/Back/Left). Our findings indicate that sighted participants can indeed process spatial information starting in their visual field (frontal 90°) and continuing in their auditory field (the remaining 270°) to create a unified image. In addition, this study replicated the results of a previous study conducted by Buchs et al. (2021), showing the efficacy of online training for visual-to-auditory SSDs. We show that subjects could even draw the stimuli within a short period of time, to some success when performing a task demanding the conversion of the entire surrounding image into a 2D visual rendering of the space (combining the back, front, and both sides onto a single 2D plane). To the best of our knowledge, this is the first time participants demonstrated projection of the back 3D space received through audio into a 2D drawn visual rendering.

Our 3D-rendered SSD employs insights derived from the growing body of knowledge on sensory substitution. The field of sensory substitution owes its beginnings to the domain of sensory rehabilitation, with research initially being conducted on conveying visual information to the blind through an alternate sense (for a review, see Maidenbaum et al., 2014). In recent years, this field has burgeoned, with several sensory substitution systems and algorithms currently being developed for various research aims. Most of these are based on the substitution of visual information through the auditory or tactile systems (Bach-y-Rita et al., 1969; Bach-y-Rita, 1983, 2004; Ptito et al., 2005; Chebat et al., 2007) and the substitution of auditory information through the tactile system (Cieśla et al., 2019, 2022), among others. Recent studies in our lab, both in the sighted (Netzer et al., 2021) and in the blind (Maimon et al., 2023), have also tested the ability to extend/augment visual-spatial perception (in both the front and the back) using auditory cues.

This study follows along these lines to provide a proof of concept for the unique system and algorithm, which builds on our prior work but takes it one step further, with the aim of pushing the limits of our current senses by providing complementary information simultaneously through other modalities. Other studies exploring such sensory enhancement include Nardini (2021) and Negen et al. (2021), who looked into integrating distance perception with an echolocation auditory type cue. Negen et al. (2021) indicated that sensory integration can become automatic, a finding with significant implications. Recently, Witzel et al. (2022) published a study exploring the automaticity of novel perceptual experiences by employing a sensory augmentation device for perceiving the north direction. These studies further support the subjective reports presented in case studies that indicate acquired automaticity and transparency following extensive use of sensory substitution devices (Ward and Meijer, 2010; Maimon et al., 2022).

As we have demonstrated in this study, subjects can perceive a shape (an abstract concept associated with the visual modality) as a combination of visual and auditory information. Our findings during the 4AFC tasks indicate that participants could use the spatial cues to heighten their success compared with monophonic renderings of the algorithm. They further suggest that fundamental advantages of the original EyeMusic, such as generalizability, remained possible and intuitive when making a move to 3D. We believe these findings are related to the fact that combining information from these modalities in our surrounding space takes place constantly, and indeed the ability to localize audio is thought to be constantly calibrated visually (Knudsen and Knudsen, 1989; Zwiers et al., 2001; Gori et al., 2014).

In our prior research, tactile inputs have also been used to show spatial awareness (Yizhar et al., 2021; Snir et al., under review). Nevertheless, in the present study, visuals and audio appear as temporally and spatially completing one another, with no overlap. The fact that this can be unified into a single visual percept strengthens the claim that spatial perception is multisensory in nature and can be recalled as such (Quak et al., 2015). This is reinforced when considering participants’ interview responses, where some indicated that the added spatial component created a more vivid and memorable experience. They also recall using spatial cues toward the reconstruction of the entire stimulus.

Further studies are warranted to see whether this could impact memory abilities in such tasks. We also believe participant accounts of using their finger to draw the stimuli through the air may provide further qualitative evidence toward a multisensory understanding of spatial information, and indeed, in this case, recruiting motor actions for the task (Clark, 2003). On the other hand, the fact that nearly half of the participants closed their eyes to better concentrate on the audio may also indicate the dominance of vision over the auditory system, as seen in various studies (Colavita, 1974; Hutmacher, 2019). Lowered activation of the auditory cortex during visual memory tasks should also be taken into consideration here (Azulay et al., 2009). Although various sensory areas have already been shown to be influenced by more than one sense (for a review, see Heimler et al., 2015 and Amedi et al., 2017), a single area representing spatial understanding has yet to be found. We believe the Vision360 technology may assist in further advancing research in this direction.

To further understand the perception of the 360° images, we employed a drawing task where we asked participants to reproduce their spatial experience onto a steady 2D plane. Although it is known in cognitive studies that spatial expressions involve some degree of ambiguity (Imai et al., 1999), in the current experiment, we utilized the structure of the experimental space (see the “Methods” section) to make clear borders between each egocentric spatial expression according to each face of the room, while maintaining the original fixed temporal sweep, in line with the EyeMusic logic. Participants made the 3D to 2D conversion intuitively and with no added training. This may be partly inherited from the scoring system, in which a missing will also lose points for shape recognition and positioning by default. Nevertheless, drawing of extra shapes would have the reverse effect, losing points for count while having no impact on the recognition and positioning scores.

Furthermore, recognition of EyeMusic stimuli may still be more challenging than spatial positioning of visuals and sound or accounting of spatially distributed objects because our healthy participants have had an entire lifetime to learn such multisensory tasks, as opposed to the conversion of temporal and auditory frequency information toward shape recognition, which they only had about an hour and a half of experience with altogether. It may nevertheless indicate multisensory spatial information as being more easily geared toward orientation than toward sensory particular information such as shape recognition. The fact that unification of the auditory and the visual information takes place at least some of the time in the majority of participants further strengthens this possibility, considering the continuous motion was presented as continuous in spatial orientation among both senses. A previous study that had a greater variety of visual shapes experienced as auditory cues tested the human ability to perceive biological movement through friction sounds produced by the action of drawing; similarly to our study, the drawings were of geometric shapes and showed the intuitive connection between kinematic movements and auditory cues (Thoret et al., 2014). This again demonstrates the multisensory connection between vision, audition, and the motor system (Jeannerod, 1995; King et al., 2009).

We use the task of drawing as a method of gauging recognition and orientation of the stimuli. The correspondence between the information provided in vision and audition and the 2D image drawn by the participants showed clear similarities. Drawings are commonly used in contemporary music to either describe or create music (Thiebaut et al., 2008). The use of drawings has also led to some interesting applications, including the development of new sonification strategies (Andersen and Zhai, 2008). Hence, drawing seemed to be a natural way of describing the motion evoked by sounds and controlling perceptually relevant attributes. Research on the blind, including a case study conducted by our lab on a blind artist, indicates an overlap between areas in the brain involved in vision and mental imagery (Amedi et al., 2008). As people can create a coherent image of their combined visual and auditory experience, it would be interesting to explore these mechanisms in the brain and see their overlap (or lack thereof). Further investigation could warrant testing for enhanced connectivity following training on Vision360, something that has been shown to occur with gradually decaying vision in adults (Sabbah et al., 2016).

Future research directions will use functional MRI to explore the possibility of novel topographies in the brain following training with sensory augmentation systems such as the Vision360 utilized in the present study. Initial research in our lab supports this idea, showing the emergence of new topographic maps following sensory substitution training and use, specifically concerning audio-rendered musical fragments similar to those used in this experiment (Hofstetter et al., 2021). Such findings may have implications for classic concepts such as the division of the brain into senses and Hubel and Wiesel’s theory of critical periods (Wiesel and Hubel, 1965). The fact that the natural perceptual capabilities can be expanded through integrating two senses well into adulthood may strengthen interpretations that call into question these two seminal theories. Yet, this matter warrants further investigation in future studies. The findings of such studies may suggest, on the one hand, that the critical periods are not as strict as has previously been accepted, and on the other that the brain is perhaps divided by tasks rather than senses, strengthening the task-specific sensory independent theory of brain development and organization (Heimler et al., 2015; Amedi et al., 2017; Heimler and Amedi, 2020). This study adds to the cumulative evidence from many studies across the last couple of decades, specifically employing sensory substitution, and perceptual cross-modal learning, the findings of which suggest that the aforementioned theories warrant revision, including the metamodal theory of brain organization (Pascual-Leone and Hamilton, 2001; Cecchetti et al., 2016) and the supramodal interpretation (Kupers and Ptito, 2011). We speculate that further findings into novel topographies in the brain resulting from training on such sensory augmentation systems would further promote this paradigm shift, and we believe our system could be employed for insights into this matter.

## Data availability statement

The datasets generated and analyzed during the current study are available in the Open Science Framework repository https://osf.io/3ntx4/.

## Ethics statement

The studies involving human participants were reviewed and approved by the Reichman University Institutional Review Board (IRB). The patients/participants provided their written informed consent to participate in this study.

## Author contributions

SS: writing—original draft, writing—review and editing, project administration, methodology, conceptualization, visualization, investigation, and formal analysis. AS: writing—original draft, writing—review and editing, methodology, conceptualization, visualization, and formal analysis. AM: writing—original draft, writing—review and editing, conceptualization, project administration, and supervision. OY: writing—original draft, writing—review and editing, methodology, conceptualization, and supervision. SH: writing—original draft, methodology, conceptualization, and investigation. KP: methodology, conceptualization, investigation, and formal analysis. AA: writing—original draft, writing—review and editing, project administration, supervision, resources, conceptualization, investigation, methodology, and funding acquisition. All authors contributed to the article and approved the submitted version.
