# Supplementary material for: Shape detection beyond the visual field using a visual-to-auditory sensory augmentation device
Source: Front Hum Neurosci. 2023 Mar 2;17:1058617. doi: 10.3389/fnhum.2023.1058617 (PMC10017858; doi:10.3389/fnhum.2023.1058617)
Supplement: Supplementary file 1 [file Data_Sheet_1.pdf]

Supplementary material

Methods

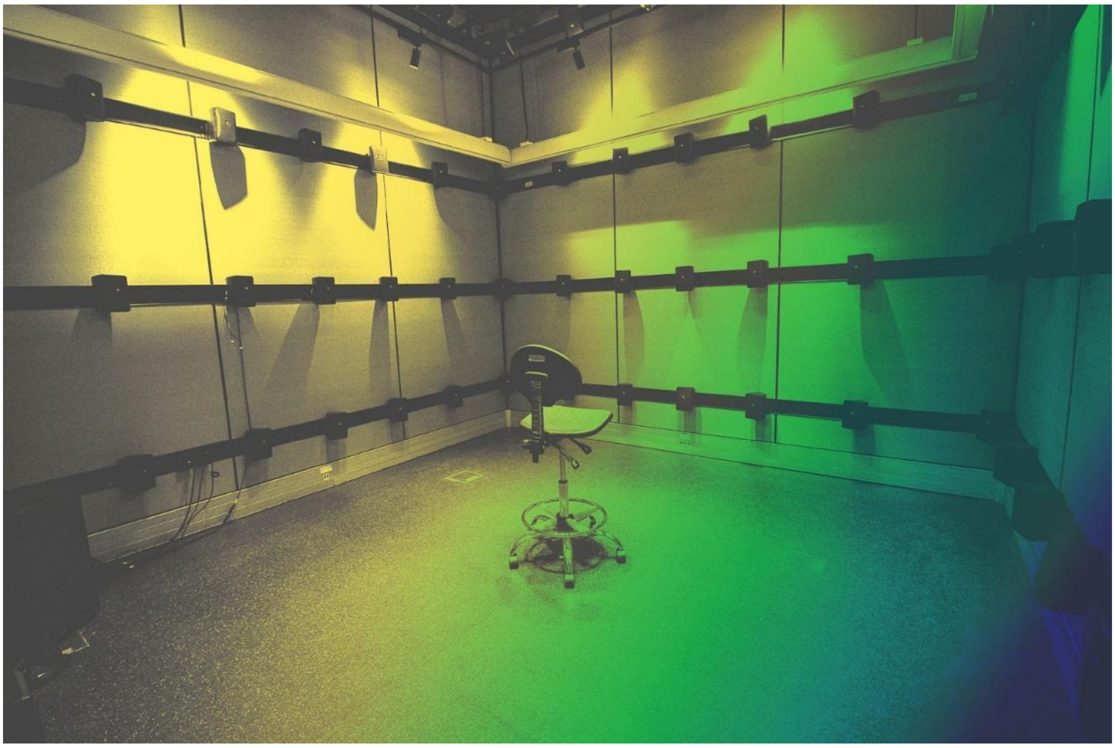

Supplementary Figure S1. A picture of the cube shaped sound proofed room in which the study took place.

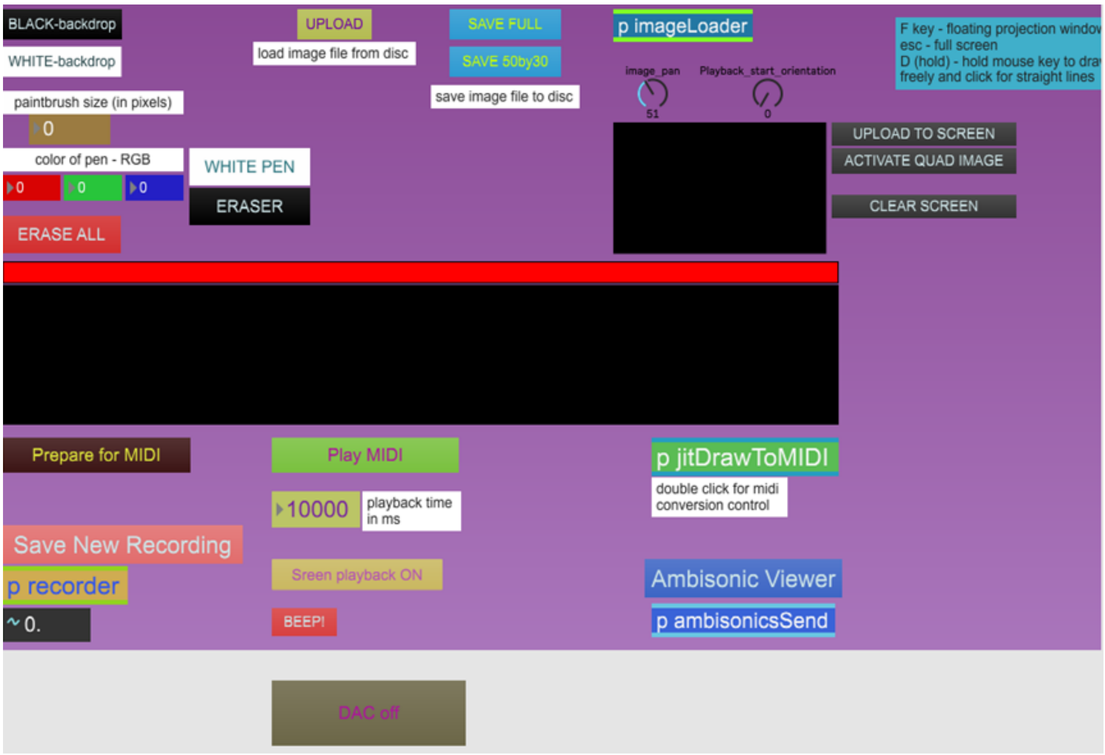

Supplementary Figure S2. The 360-degree Audio-visual transformation algorithm interface.

Stimuli. all stimuli used in the present study comprised of “EyeMusic”, white pixels with black backdrop shapes (https://osf.io/3ntx4/files/osfstorage).

Basic at home training. an online training platform was created for the purpose of this experiment (EyeMusic Training (musing-goldwasser-4d5e1c.netlify.app)).

In laboratory pre-test. using the dedicated online training platform, an online pre-test form was constructed (https://dreamy-nobel-c92a97.netlify.app/).

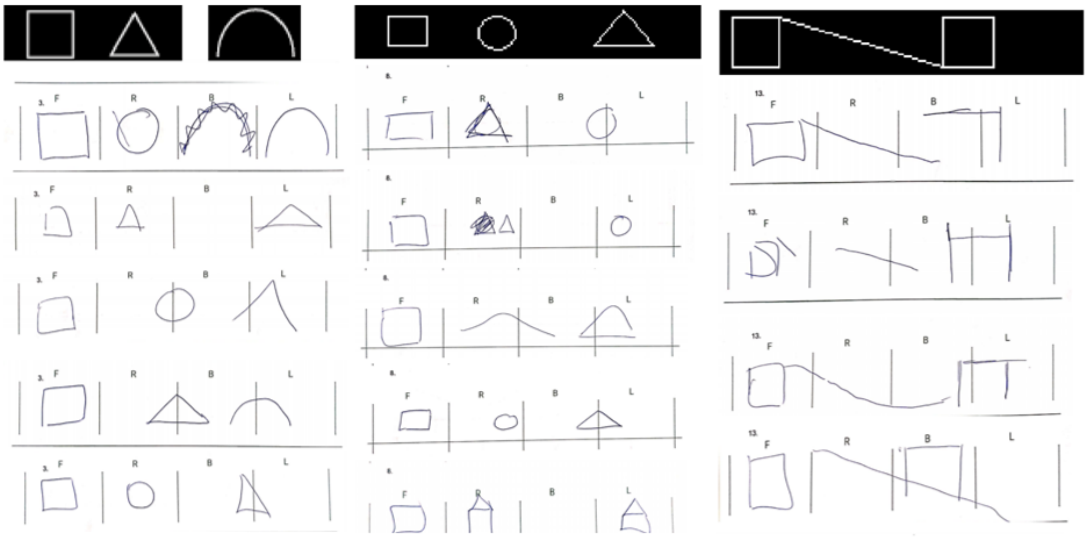

Supplementary Figure S3. an example of stimulus drawn by participants.

Participants Drawings. all participants’ drawings can be found in (https://osf.io/3ntx4/files/osfstorage/63049d0f80b0d81af6fe93f0).

Results

Drawings Task - Phase 3 - Group score

The group score for each of the measures within each category was calculated by summing each participant’s stimuli scores within the measure, then averaged among the group and converted to percentages.
For individual results per stimuli see file “Coding drawings individual results per stimuli” in the osf - https://osf.io/3ntx4.

Category 1 (stimuli containing separated trained shapes)
(max result = 6, Unification max result = 3)

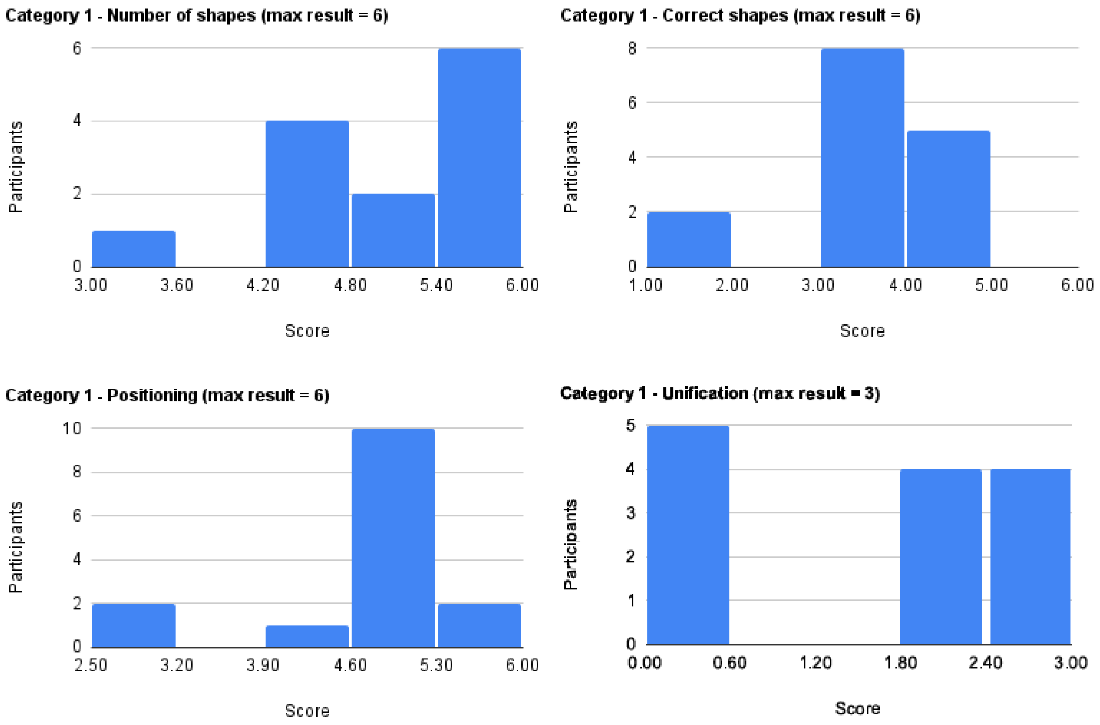

Supplementary Figure S4. Histograms of group score results for category 1. Scores indicate the sum of all stimuli measures within category 1.

Category 1 (stimuli containing separated trained shapes)
(max result = 6, Unification max result = 3)

Participant #	Num of Shapes	Correct Shapes	Positioning (max = 6)	Unification
1	5.66	4.49	5.25	2
2	4.66	3.24	4.66	0
3	4.86	3.24	4.99	0
4	6	4.5	5.08	3
5	5.66	1.91	5.41	2
6	4.5	3.49	4.66	1
7	5.41	3.24	4.74	2
8	5.16	3.24	4.82	0

**9**	4.74	3.08	2.91	2
**10**	5.75	4.24	5.16	3
**11**	4.32	3.41	4.66	0
**12**	5.75	4.74	4.91	3
**13**	3.07	1.33	2.83	0
**14**	5.5	3.41	4.41	1
**15**	6	4.74	5.66	3
Average	5.13	3.49	4.68	1.46
SD	0.79	0.97	0.8	1.24
	**85.60%**	**58.00%**	**78.00%**	**46.70%**

Category 2 (extended single shapes)
(Overall max result = 2)

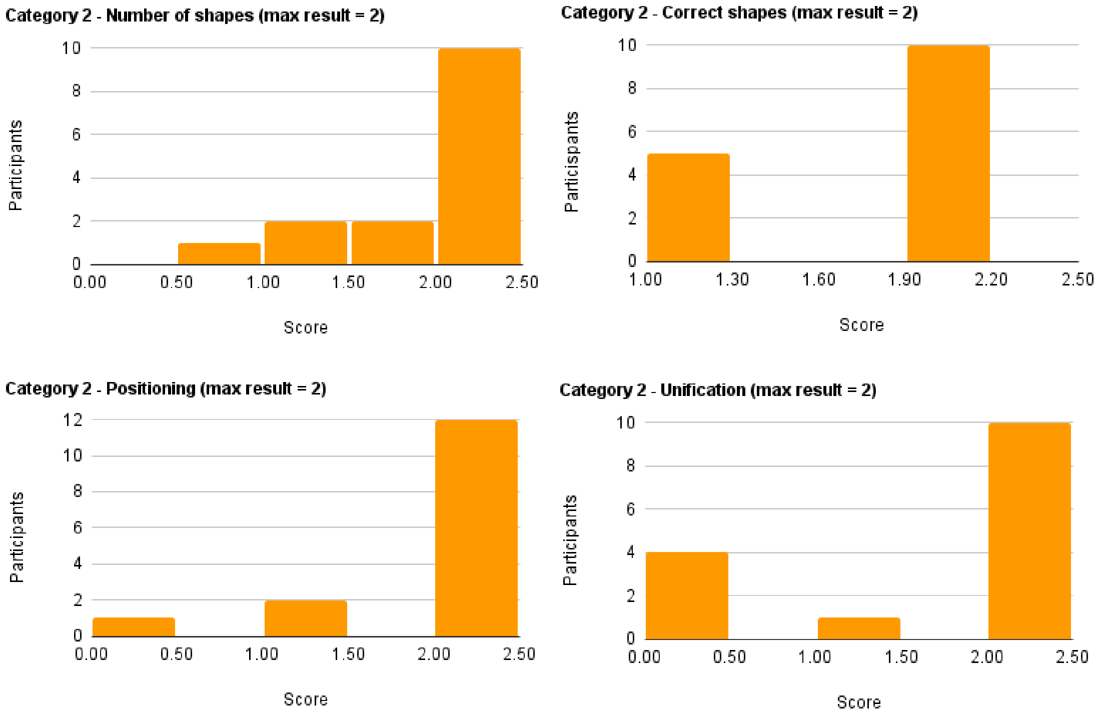

Supplementary Figure S5. Histograms of group score results for category 2. Scores indicate the sum of all stimuli measures within category 2.

<u>**Participant #**</u>	<u>**Num of Shapes**</u>	<u>**Correct Shapes**</u>	<u>**Positioning**</u>	<u>**Unification**</u>

frontiers

**1**	2	2	2	2
**2**	1.5	1	2	0
**3**	2	2	2	2
**4**	2	2	1	2
**5**	1	1	0	1
**6**	1.5	1	2	0
**7**	2	2	2	2
**8**	0.5	1	1	0
**9**	2	2	2	2
**10**	2	2	2	2
**11**	2	2	2	2
**12**	2	2	2	2
**13**	1	1	2	0
**14**	2	2	2	2
**15**	2	2	2	2
Average	1.7	1.6	1.7	1.4
SD	0.49	0.49	0.59	0.9
	**85%**	**83%**	**86.7%**	**70%**

Category 3 (combining extended and trained shapes in tandem)
(Overall max result = 5)

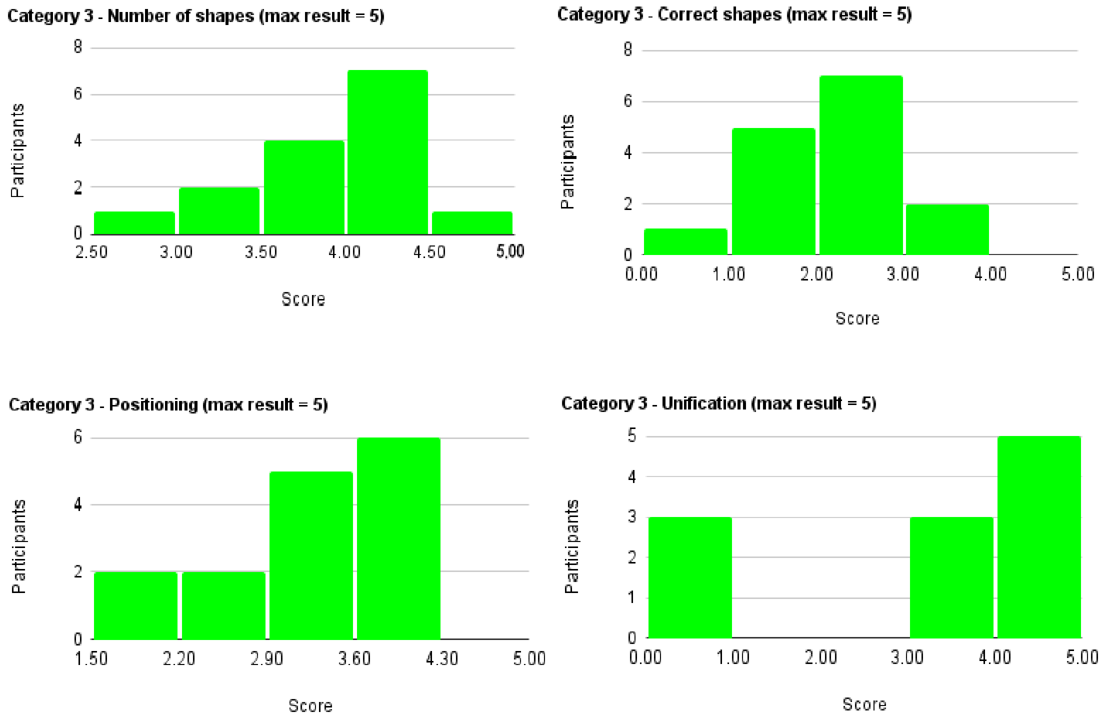

Supplementary Figure S6. Histograms of group score results for category 3. Scores indicate the sum of all stimuli measures within category 3.

Participant #	Num of Shapes	Correct Shapes	Positioning	Unification
**1**	4.01	2.32	4.16	3
**2**	3.92	1.91	2.66	0
**3**	4.01	2.57	3.66	3
**4**	4.26	2.57	3.66	5
**5**	3.67	1.41	2.49	5
**6**	4.26	1.41	3.32	4
**7**	4.21	2.57	3.32	5
**8**	3.39	1.24	2.07	0

**9**	3.66	3.5	4.25	3
**10**	4.01	2.57	3.32	4
**11**	3.34	1.74	3.41	4
**12**	4.8	3.41	4.25	4
**13**	2.6	0.33	1.91	0
**14**	3.87	2.82	3.66	5
**15**	4.26	2.41	3.25	4
Average	3.88	2.18	3.29	3.26
SD	0.51	0.85	0.73	1.8
	77.7%	43.7%	66%	65.3%

References

SHVADRON, S. (2022, August 23). Vision360. Retrieved from osf.io/3ntx4.
DOI 10.17605/OSF.IO/3NTX4.
